# Phosphodiesterase-4 Inhibitor Roflumilast-Mediated Protective Effect in Sepsis-Induced Late-Phase Event of Acute Kidney Injury: A Narrative Review

**DOI:** 10.3390/ph15070899

**Published:** 2022-07-20

**Authors:** Imran Kazmi, Fahad A. Al-Abbasi, Muhammad Afzal, Muhammad Shahid Nadeem, Hisham N. Altayb, Gaurav Gupta

**Affiliations:** 1 Department of Biochemistry, Faculty of Science, King Abdulaziz University, Jeddah 21589, Saudi Arabia; fabbasi@kau.edu.sa (F.A.A.-A.); mhalim@kau.edu.sa (M.S.N.); hdemmahom@kau.edu.sa (H.N.A.); 2 Department of Pharmacology, College of Pharmacy, Jouf University, Sakakah 72341, Saudi Arabia; afzalgufran@ju.edu.sa; 3 School of Pharmacy, Suresh Gyan Vihar University, Mahal Road, Jagatpura 302017, Jaipur, India; drgaurav.gupta@mygyanvihar.com; 4 Department of Pharmacology, Saveetha Dental College, Saveetha Institute of Medical and Technical Sciences, Saveetha University, Chennai 602105, Tamil Nadu, India; 5 Uttaranchal Institute of Pharmaceutical Sciences, Uttaranchal University, Dehradun 248007, Uttarakhand, India

**Keywords:** PDEs, roflumilast, S-AKI, inflammation, sepsis

## Abstract

Severe infections such as viral, bacterial, or fungal sepsis can cause an inflammatory response in the host, leading to organ failure and septic shock—phosphodiesterase-4 (PDE-4) inhibiting related agents from suppressing cyclic adenosine monophosphate (cAMP) degradation. Regulatory organisations have approved some substances in this category to reduce the risk of chronic obstructive pulmonary disease (COPD) exacerbations in patients with chronic bronchitis and a history of COPD exacerbations. Roflumilast has been shown to alleviate inflammatory responses, thus regulating airway inflammation. Additionally, roflumilast therapy dramatically enhanced B-cell lymphoma 2 (Bcl-2) expression, an anti-apoptotic marker lowered in septic animals. Previous research has indicated that roflumilast may help reverse sepsis-induced liver and lung harm, but whether it is also effective in reversing sepsis-induced renal impairment remains unknown. Therefore, this review determines whether roflumilast protects against renal dysfunction, inflammatory response, and apoptosis in sepsis-induced kidney damage. Additionally, we discussed the molecular mechanism through which roflumilast exerts its protective effect to uncover a possible treatment agent for sepsis-induced renal impairment.

## 1. Introduction

Phosphodiesterase-4 (PDEs), which break down cAMP by hydrolysing the phosphodiester link of cAMP to form AMP, and adenylate cyclases, which catalyse the cyclization of adenosine triphosphate (ATP) into cAMP, both play a role in tightly managing intracellular cAMP levels. Eleven people represent the PDE family. PDEs 4, 7, and 8 hydrolyze cAMP, whereas PDEs 1, 2, 3, 10, and 11 hydrolyze cAMP and cyclic guanosine monophosphate (cGMP). PDEs 5, 6, and 9 hydrolyze cGMP. The most prominent mammalian PDE family members, PDE4 A through D, are encoded by four genes, with more than 25 splice variants produced by combining the four genes. PDE4 isotypes are extensively distributed throughout the body, except in the heart, where they are absent or weakly expressed but are substantially expressed in skeletal muscle, nerve, and inflammatory cells [1,2,3,4].

Some of the substances from this category have been licenced by regulatory authorities to reduce the risk of COPD exacerbations in patients with chronic bronchitis and a history of COPD exacerbations [5,6,7]. PDE4 can be a therapeutic target for a wide range of diseases. Antidepressant benefits and improved cognition and memory have been demonstrated in animal models with Rolipram, the most extensively investigated generic PDE4 inhibitor [8,9]. In addition, the anti-inflammatory characteristics of PDE4 inhibitors benefit their use [10,11,12]. Since modifying the metabolic benefits of resveratrol, Rolipram can activate AMP-activated protein kinase (AMPK) and increase mitochondrial activity, thereby preventing obesity and improving glucose tolerance in mice fed high-fat diets, as demonstrated in this study [13,14,15]. Whether roflumilast’s capacity to activate AMPK and Sirtuin 1 (SIRT1) in the kidneys is specific to PDE4 inhibitors or not is unknown.

**Figure 1:**
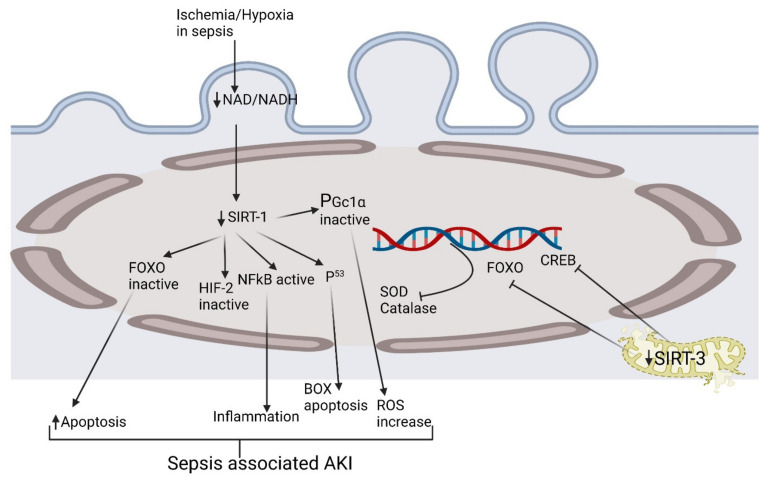
Exploring the pathophysiological activation of various biomolecular pathways in septic acute kidney injury. CREB, cAMP-response element-binding protein; HIF-2, hypoxia-inducible factor-2; FOXO, forkhead box O; NAD, nicotinamide adenine dinucleotide; NF-κB, nuclear factor kappa-light-chain-enhancer of activated B cells; PGc1α, peroxisome proliferator-activated receptor-gamma coactivator (PGC)-1alpha; ROS, reactive oxygen species; SIRT1, Sirtuin 1; SODs, Ssuperoxide dismutases.

As part of our ongoing investigation into the cellular roles and activities of AMPK, PDE4, and Sirt1, many compounds have been examined for their capacity to inhibit PDE4. However, research has indicated that roflumilast may help reverse sepsis-induced liver and lung harm, but whether it effectively reverses sepsis-induced renal impairment remains unknown. Therefore, this review determines whether roflumilast protects against renal dysfunction, inflammatory response, and apoptosis in sepsis-induced kidney damage (Figure 1). Additionally, we discussed the molecular mechanism through which roflumilast exerts its protective effect to uncover a possible treatment agent for sepsis-induced renal impairment.

## 2. Selection of Literature Review

Medline, Mendeley, PubMed, ScienceDirect, SpringerLink, and Google Scholar were used to search for studies that could be relevant. We used various terms to perform the literature search, alone and in combination. As a part of our research, the following terms were used: ‘Definition sepsis-associated acute kidney injury, ‘Epidemiology S-AKI, ‘Early-stage sepsis-associated inflammation’, ‘Pathogenesis of S-AKI’, ‘Involvement of various biomarkers of S-AKI, ‘Cytokine activation mediated biological response in S-AKI, ‘Evidence-based roflumilast treatment in S-AKI’, ‘Role of kidney injury molecule (KIM) and N-acetyl-*β*-D-glucosidase (NAG) in kidney injury. Only works in English were considered for this investigation. The reference lists of the papers identified have also been checked for items not detected by an initial search method.

## 3. Clinical and Preclinical Events of Septic Acute Kidney Injury

Severe infections such as viral, bacterial, or fungal sepsis can cause an inflammatory response in the host that leads to organ failure and septic shock. “Life-threatening organ failure induced by an altered host immunological responsiveness to infection” was the new definition of sepsis in 2016 [16]. Septic shock is a kind of sepsis in which the underlying circulatory and cellular/metabolic abnormalities are severe enough to increase mortality. Throughout sepsis, the kidney is among the first organs to be affected. AKI and sepsis syndrome are complicated and varied illnesses with many risk factors. AKI is the primary cause of mortality and disability in ICUs in developed countries [17,18]. There is no one effective therapy for AKI, despite advancements in supportive care, such as infection control and the use of blood products. The mainstay of treatment for kidney disease is early identification and appropriate supportive and preventative maintenance. There have been various analyses of the epidemiological data of sepsis-related acute renal injury.

Furthermore, the death rate for adults with AKI during sepsis is 60% [19], and the figure for children ranges from 57% to 66% [20]. Sepsis-related AKI is more severe than nonseptic AKI and is linked with a higher fatality rate. S-AKI was found in 48.1 per cent of patients overall, with 59.2 per cent of those in the ICU and 31.6 per cent of those outside the ICU, according to a recent study published in dove press and a 55percent overall fatality incidence among S-AKI patient populations. In addition, AKI in sepsis is associated with the rapid decline in renal function resulting in nitrogenous waste substance retention, a common symptom of sepsis [21,22]. Even though AKI complicates the short-term treatment of patients with sepsis, it also increases the risk of long-term consequences, namely, the development of end-stage renal disease (ESRD), kidney failure with replacement therapy (KFRT), and short- and long-term fatalities [23,24,25,26]. As with sepsis, ongoing efforts to standardise the definition of AKI have resulted in the development of the following three major classification systems over the last two decades: the acute dialysis quality initiative (ADQI)—proposed Risk, Injury, Failure, Loss of kidney function, and RIFLE criteria, the AKIN criteria [27], and the most recent KDIGO criteria [28]. To determine the diagnosis of AKI, all three categorization systems rely on a rise in serum creatinine (sCr) and a reduction in urine output (UO). Despite this, current sepsis recommendations continue to propose using the sequential organ failure assessment (SOFA) score to identify AKI, which is problematic since SOFA makes no distinction between chronic and acute renal illness or takes demographic disparities in baseline sCr level into account.

In general, the previous murine model divides sepsis into an initial stage of hyperinflammatory/immune activation (around 0–6 h) and a late episode of immunological suppression (24–48 h) [29,30]. During the initial stages of sepsis, pro-inflammatory cytokines such as tumour necrosis factor α (TNFα), interleukin-1 (IL-1), IL-2, IL-6, IL-8, and interferon-gamma (IFN-γ) directly exacerbate the damage by attracting diverse leukocytic populations to the site of injury, resulting in a vicious cycle of the inflammatory process [31]. An increase in pro-inflammatory mediators released into the body, which are then filtered by the kidneys, leads to damage to the kidney’s proximal tubules and the loss of tubular epithelial cells, leading to metabolic irregularities and kidney injury as a result of this stimulus [32,33].

In the initial stages of S-AKI, the hyperinflammatory and active immunological responses, together with the oxidative load, promote further pathological alterations such as cellular proliferation, podocyte injury, extracellular matrix (ECM) deposition, and activation of pro-apoptotic pathways [34]. The immunosuppression seen in the late stages of sepsis compromises the body’s defences, leading to enhanced organ damage. Interestingly, prolonged inflammation and poor T cell response were observed in old septic mice and geriatric septic humans 24 h after infection (late-stage) and were linked to decreased life expectancy [35]. The increased number of monocytes and neutrophils found in the peripheral blood of old mice and humans throughout a comparable period suggests that the immune system is out of whack in the late stages of sepsis. Septic patients in the later or immunosuppressive period (three to five days) have been shown to have high CD10 and CD16 and altered chemotaxis of neutrophils in their blood, which raises the risk of mortality [36].

## 4. Recent Updates of Biomolecules in S-AKI

Galactin-3 (Gal-3), a soluble β-galactoside-binding substance, is extensively expressed in the cardiac, renal, hepatic, pulmonary tissues, and intestine and is particularly abundant in activated macrophages and mast cells and basophils [37]. The principal renal Gal-3 synthesis and secretion sources are inflammatory cells and macrophages. Gal-3 expression in the kidney is quickly increased in models of renal injury [38,39,40,41,42]. According to recent research, increased Gal-3 levels after ICU admission predicted S-AKI and death in sepsis patients, but Gal-3 inhibition in a CLP rat model dramatically decreased S-AKI and mortality. S-AKI and sepsis mortality may be linked to Gal-3’s participation in the aetiology of S-AKI [43].

To maintain renal homeostasis and sustain the metabolic pressure seen during sepsis, the existence of healthy mitochondria is required [44,45,46]. Renal ATP depletion and elevated production of reactive oxygen species (ROS) are caused by mitochondrial failure in sepsis and eventually result in cell homeostasis collapse and organ dysfunction [47]. When sepsis develops, inflammatory molecules such as TNF-α and IL-6 may bind to signal transducers and activators of transcription 3 (STAT3) due to early inflammation in the body. As a result, STAT-3 is activated, enhancing its transcriptional capacity for IL-6 and TNF-alpha. This may eventually convey pro-inflammatory signals and worsen inflammation. As indicated in muscle biopsies, sepsis reduces ATP levels and increases biomarkers for mitochondrial dysfunction. It reduces antioxidant defense in muscle samples from 16 critically ill patients compared to 10 healthy, age-matched people having elective hip surgery [48].

**Figure 2:**
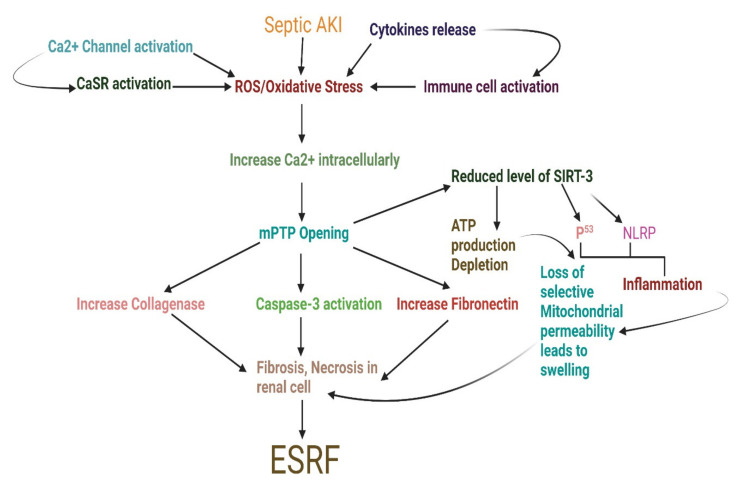
Schematic diagram exploring sepsis-associated acute kidney injury progression through enhanced biomolecules burden to End-stage renal failure. AKI, acute kidney injury; ATP, adenosine triphosphate; CaSR, calcium-sensing receptor; ESRF, end-stage renal failure; mPTP, mitochondrial permeability transition pore; NLRP, NACHT, LRR, and PYD domains-containing protein; ROS, reactive oxygen species; SIRT3, sirtuin 3.

On the other hand, mitochondrial biogenesis is linked with an enhanced chance of longevity in septic shock, and pathological processes of sepsis-AKI indicate the crucial function of healthy mitochondria in renal recovery and lifespan [49,50,51,52,53]. In addition, participants with sepsis-AKI showed significantly lower mRNA signaling of SIRT1, which is implicated in regulating oxidative stress and biogenesis, than control participants [54]. In the case of pulmonary hypertension, activation of AMPK also slowed the growth of Gal-3-activated cells [55]. Additionally, when all these active biological components are connected, Sirtuin 1 can be activated indirectly via AMPK by increasing the availability of nicotinamide adenine dinucleotide (NAD+) in the setting of kidney injury. Protection against AMPK activation in the CLP model was related to enhanced sirtuin 1 expression [56,57], corroborating our findings. These studies consistently demonstrate that activating AMPK during sepsis protects against AKI and lowers mortality, independent of the underlying molecular mechanism [57]. Both AMPK and SIRT1 regulate each other and share several target molecules, and this review will assess the evidence that these similarities are due, at least in part, to reciprocal regulation (Figure 2).

## 5. Evidence of Roflumilast at the Molecular Level for Acute Kidney Disease in Sepsis

**Figure 3:**
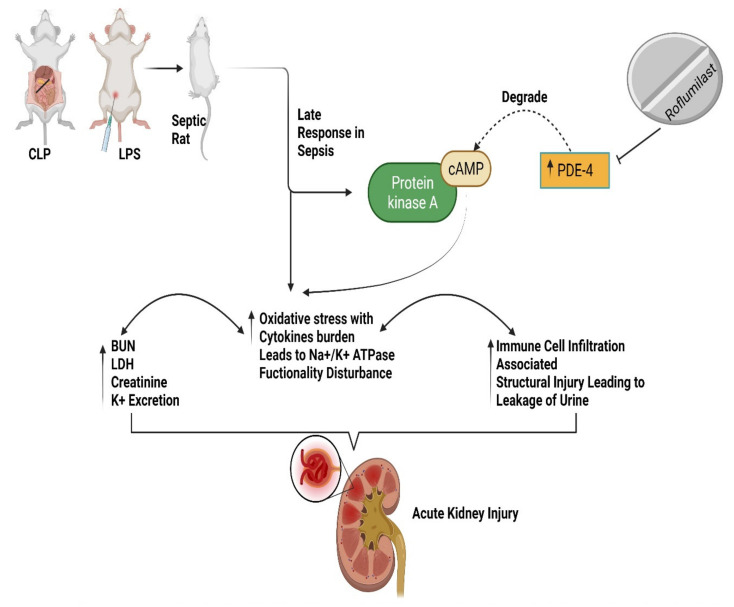
Represents septic rats with PDE-4 inhibition leading to increased cAMP levels, alleviating renal impairment, oxidative and inflammatory stress, and decreasing immune cell infiltration and leakage in urine [15]. BUN, Blood urea nitrogen; cAMP, cyclic adenosine monophosphate; cecal ligation and puncture (CLP), LDH, lactate dehydrogenase; lipopolysaccharide (LPS); PDE-4, phosphodiesterase-4.

The recruitment of inflammatory cells increases ROS generation at the damage site, aggravating renal impairment [58]. Free radicals activate inflammatory cells such as neutrophils and macrophages, promoting the generation of additional inflammatory mediators and uncontrolled ROS accumulation, thus establishing a vicious cycle of inflammatory responses and oxidative load via the nuclear factor kappa B (NF-κB) pathway [59]. Research indicated that the PDE-4 blocker roflumilast reduced lipopolysaccharides (LPS)-associated nitric oxide (NO), TNF-alpha, and IL-1beta expression in the macrophage cell line RAW 264.7 by lowering NFk-B stimulation, stress-activated protein kinases (SAPK)/Jun amino-terminal kinases (JNK), and p38 MAPK mechanisms [60]. Furthermore, the research results indicated that following administration of a PDE-4 inhibitor derivative, Sirt1, phosphoinositide 3-kinases (PI3Ks), and pAKT protein expression rise and renal function is effectively maintained. Injured neurons treated with PDE4 inhibitors exhibit increased Sirt1 expression, AKT phosphorylation, and decreased apoptosis [61]. Septic rats had a high bacterial load, myeloperoxidase activity, decreased cAMP concentration, increased vascular permeability, lowered Na + K + ATPase activity, increased caspase-3, excessive interstitial leukocyte infiltration, elevated renal expression of PDE-4B and 4D isoforms, and electrolyte imbalance in addition to histological changes. A large dosage of roflumilast pretreatment avoided all AKI-related symptoms in septic rats [62]. The activation of PDE-4 isoforms strongly contributes to the inflammatory response and the engagement of the immunological system. Inflammatory mediators such as TNF-α may be released by PDE-4B, one of the PDE-4 isoforms, on monocytes and neutrophils [63]. PDE-4B mutant mice could not secrete TNF-α from circulated leukocytes in response to LPS, revealing that this isoform plays a pivotal role in the pathogenesis of inflammatory disorders [64]. Additionally, suppression of PDE-4B rectified inflammatory cytokine generation and death in kidney tubular epithelial cells following cisplatin therapy. Other research reveals increased expression of PDE-4B and PDE-4D variants in the bronchiolar lavage fluid of treated animals with ovalbumin; however, inhibiting the PDE-4B subtype significantly cured rodent allergies [65]. Roflumilast at a large concentration significantly decreased the production of PDE-4B and PDE-4D subtypes, indicating that both variants significantly impact the sepsis AKI condition. A study found that activating the cAMP–CREB pathway decreased cytokine production and promoted inflammation [66]. These results demonstrate that silencing both variants was adequate for elevating cAMP concentrations in kidney homogenates and producing reno-protective benefits in the S-AKI model. Additionally, pre-treatment dosage decreased renal oxidative stress and inflammatory cytokine production in CLP rats’ renal homogenates. Additionally, this therapy inhibited the activity of myeloperoxidase (MPO) in kidney homogenates. MPO, a neutrophil-secreted enzyme, exhibits significant pro-oxidant and pro-inflammatory activity [67]. The MPO activity in the urine and plasma of S-AKI patients rose 24 h after neutrophil activation was boosted by IL-8, according to Törnblom and colleagues [68]. As a consequence of ROS overproduction, various cellular processes, including the polyol pathway, the MAPK, AMPK, and the NF-kB signaling pathways, as well as transcription factors such as activator protein 1 (AP-1), nuclear factor erythroid 2–related factor 2 (Nrf2), and forkhead box O (FOXO) are activated [69,70]. Our research also revealed that the serum of cecal ligation and puncture (CLP) septic rats had higher lactate dehydrogenase (LDH) activity, a sign of impaired aerobic respiration. Increased baseline lactate levels in sepsis were related to decreased renal microcirculation, hypoxia, and cellular dysmetabolism [71,72]. Sepsis causes an increase in anaerobic glycolysis and a buildup of lactate due to reduced renal perfusion and mitochondrial dysfunction [73]. This results in intracellular hypoxia, as most oxygen is used during anaerobic glycolysis to create ATP from lactate. Thus, renal tissue ATP levels fall short of supporting the activity of ATP-dependent transporters and enzymes in nephron segments. According to another study, CBU91 is a strong and selective PDE4 inhibitor that increases mitochondrial activity in myotubes while activating Sirt1 and AMPK [14]. Notably, AMPK and SIRT1 act synergistically and have comparable impacts on various biological functions, including DNA repair, cell proliferation, mitochondrial function, and cell metabolism [74]. For example, AMPK enhances SIRT1 activity by raising the intercellular NAD+ concentration [75]. Alternatively, SIRT1 deacetylates and activates liver kinase B1 (LKB1), AMPK’s upstream kinase, activating AMPK and suppressing ROS generation [76,77]. SIRT1 and AMPK have several shared targets, including FOXOs, the peroxisome proliferator-activated receptors (PPARs), and peroxisome proliferator-activated receptor-gamma coactivator (PGC). Joint activation of SIRT1 and AMPK can activate these downstream effectors independently or in combination, exerting antioxidant protective responses and alleviating oxidative stress [15]. In this experiment, pre-treatment with a PDE-4 inhibitor significantly reduced LDH levels, showing that this medication is beneficial in addressing metabolic imbalance and tissue hypoperfusion associated with septic AKI (Figure 3).

In prior research, roflumilast therapy ameliorated diabetic nephropathy in rats by restoring kidney function, reducing oxidative stress, and accumulating extracellular matrix, minimizing glomerular damage and apoptosis [15]. Furthermore, apoptosis, a controlled cell death, is critical for the damage and growth of multicellular organisms across various tissues [78]. A combination of inflammation and apoptosis may influence the development of AKI in sepsis. This study observed that CLP enhanced the number of apoptotic cells, but roflumilast therapy reversed this effect. Additionally, roflumilast treatment dramatically improved Bcl-2 expression, an anti-apoptotic marker lowered in septic animals [79].

On the other hand, roflumilast had the opposite effect on cleaved caspase-3, cleaved caspase-9, and Bax, all of which were increased in septic mice, indicating that roflumilast may protect animals against kidney damage in part by inhibiting cell death [80]. The p38 MAPK phosphorylation was likewise dramatically lowered by Roflumilast. The roflumilast intervention improved the tissue morphology of sepsis mice by reducing JAK and STAT-3 protein and mRNA expression [81] and, making things even more interesting, roflumilast significantly decreased the activity of STAT3, as well as Janus kinase 1 and Janus kinase 2, which are both downstream of STAT3. Furthermore, phosphorylation of p38 MAPK by interferon-gamma and TNF-alpha in HaCaT cells was dramatically inhibited by cAMP-dependent PKA activity [82,83]. It seems that roflumilast guards against sepsis by suppressing STAT3, MAPK, and NF-kB signalling pathways [67].

KIM1 and neutrophil gelatinase-associated lipocalin (NGAL) expression levels are the primary markers of early AKI, and their monitoring is critical for guiding AKI prognosis. KIM1, an extracellular and cytoplasmic domain, is expressed at deficient levels in normal kidneys [84]. Extracellular fragments may cleave and enter tubule lumens after kidney damage, which may be detected in the urine. A characteristic of renal tubular impairment is the presence of NGAL [85]. Severe sepsis patients may benefit from using NGAL as a diagnostic and predictive biomarker for AKI because of its high sensitivity and low negative predictive value [86,87]. Additionally, the study’s experimental results indicated that roflumilast could decrease the expression of KIM1 and NGAL, which were raised in sepsis-induced AKI, implying that roflumilast acted as a protective factor for kidney function. The pathological alterations associated with sepsis-induced AKI in the kidney are thought to be strongly connected to the course of the disease, as evidenced by the histological findings in various investigations [88].

For individuals with COPD, the recommended dose of roflumilast is 500 g/day. For the first time, roflumilast has been shown to protect mice against CLP-caused sepsis, with a statistically significant level of protection at 1.0 mg/kg. Roflumilast dosed at 1.0 mg/kg in mice is comparable to 0.08 mg/kg in humans, as determined by the FDA’s “Conversion of Animal Doses to Human Comparable Doses” [89,90,91]. Although roflumilast is often given in the range of 1–10 mg/kg in animal studies, it is worth noting that the study’s minimum effective dose of roflumilast is almost tenfold that of the recommended human dosage for COPD.

## 6. Conclusions

PDE-4 inhibitors and roflumilast decreased S-AKI by lowering immune cell infiltration into renal tissues and urine leakage, as observed by us. In addition, researchers found that Roflumilast influenced antioxidant and inflammation levels and Na + K + ATPase activity through the upregulation of cAMP levels in kidney tissue homogenates for these immunosuppressive effects. According to the reviewed literature, pre-treatment with Roflumilast enhanced kidney function and decreased histological deterioration in septic rats’ renal tubules. Roflumilast, a PDE-4 inhibitor, has been shown to defend against S-AKI in an animal model of septic shock, and this new information sheds light on how it works.

## Data Availability

Data sharing not applicable.
